# Number-Based Visual Generalisation in the Honeybee

**DOI:** 10.1371/journal.pone.0004263

**Published:** 2009-01-28

**Authors:** Hans J. Gross, Mario Pahl, Aung Si, Hong Zhu, Jürgen Tautz, Shaowu Zhang

**Affiliations:** 1 Chair of Biochemistry, Biocentre, University of Würzburg, Würzburg, Germany; 2 BEEgroup, Biocentre, University of Würzburg, Würzburg, Germany; 3 ARC Centre of Excellence in Vision Science, Research School of Biological Sciences, The Australian National University, Canberra, Australia; Max-Planck-Institut fuer Neurobiologie, Germany

## Abstract

Although the numerical abilities of many vertebrate species have been investigated in the scientific literature, there are few convincing accounts of invertebrate numerical competence. Honeybees, *Apis mellifera*, by virtue of their other impressive cognitive feats, are a prime candidate for investigations of this nature. We therefore used the well-established delayed match-to-sample paradigm, to test the limits of honeybees' ability to match two visual patterns solely on the basis of the shared number of elements in the two patterns. Using a y-maze, we found that bees can not only differentiate between patterns containing two and three elements, but can also use this prior knowledge to differentiate three from four, without any additional training. However, bees trained on the two versus three task could not distinguish between higher numbers, such as four versus five, four versus six, or five versus six. Control experiments confirmed that the bees were not using cues such as the colour of the exact configuration of the visual elements, the combined area or edge length of the elements, or illusory contours formed by the elements. To our knowledge, this is the first report of number-based visual generalisation by an invertebrate.

## Introduction

The numerical ability of non-human animals has long been a source of fascination and contention to members of the academic and lay communities alike. As early as the 1940s, Otto Koehler and his students were able to demonstrate that pigeons could be trained to peck at a cluster of seeds containing, for example, exactly three seeds, and ignore the cluster containing two. Jackdaws could also learn a matching-to-sample paradigm to recognise visual patterns with the “correct” number of dots, and obtain a hidden food reward [1]. Drawing inspiration from these humble beginnings, later researchers have been able to show that a wide range of vertebrate species (such as racoons [2], dolphins [3], monkeys [4], songbirds [5] and even salamanders [6]) also possess some form of numerical competence. Indeed, studies on chimpanzees have uncovered the impressive ability of this species to order numerosities on a scale, even in the absence of a language faculty [7].

However, even a brief survey of the literature on animal numerical abilities will reveal a surprising asymmetry: mostly vertebrate species have been studied to date, leaving the numerical cognition of invertebrates largely unexplored. We intend to correct this imbalance in this paper, by reporting our novel findings on the numerical ability of the honeybee. Research in the last two decades has shown that honeybees possess impressive cognitive abilities, such as the capacity to match and categorise visual objects [8], learn the concept of sameness and difference [9], associatively group and recall visual objects [10], and carry out different tasks within a temporal context [11]. Indeed, an early claim that honeybees might be able to distinguish between flowers of different species by ‘counting’ the number of petals [12], was probably confounded by the insect's ability to detect bilateral symmetry [13], [14] and categorize visual objects by their overall shape [8]. While it would be unwise to expect honeybees to perform tasks comparable to those attributed to chimpanzees, we thought it not unreasonable to expect at least a rudimentary form of numerical ability in this insect. After all, an estimate of the number of flowers visited on a foraging trip, weighed against the amount of nectar collected, could yield an estimate of the profitability of a food source [15]. At least one study has hinted at the possibility that foraging honeybees might be able to remember the number of landmarks encountered on the way to a food source [16]. Bees could also be trained to match either the ‘first’ or ‘second’ sample pattern in a sequence of two, to the correct choice pattern [17]. Finally, a recent variant of the Chittka and Geiger study reported sequential counting of landmarks by bees flying to a food source [18].

We therefore set out to determine if any form of numerical cognition could be attributed to the honeybee. Using a y-maze setup, and a delayed-match-to-sample (DMTS) paradigm, we trained honeybees to make generalisations about the number of elements in a visual pattern, and distinguish between arrays composed of two and three elements. Having controlled for lower-order cues such as area and edge length, we find that the bees were using the number of elements in each pattern as a cue on which to base their decisions. While our results neither suggest that bees can ‘count’, nor that they can order numerosities, we believe that this is the first report of number-based visual generalisation in an invertebrate.

## Results

The experiments were carried out repeatedly several times in Germany and in Australia during 2006 to 2008.

### Training on the basic DMTS task

**Figure 1:**
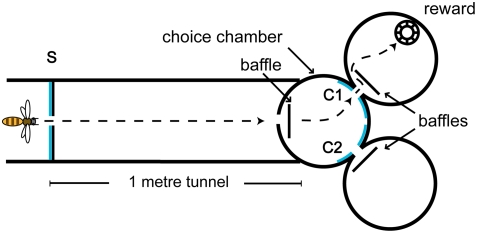
Layout of the Delayed Match-to-Sample (DMTS) experimental apparatus. The bee encounters and flies through the initial sample pattern (S) before traversing a 1m-long tunnel with a perspex roof. There is a baffle behind the entrance of the decision chamber and baffles behind the entrances of the choice chambers The baffles prevented the bees from experiencing the stimuli in the decision chamber until they had entered it, and from viewing the feeder from the decision chamber. Upon entering the choice chamber, she is presented with two choice patterns (C1 and C2), only one of which (C1 in this case) has the same number of dots as S. The bee must choose the matching pattern C1 in order to obtain a hidden reward of sugar solution.

**Figure 2:**
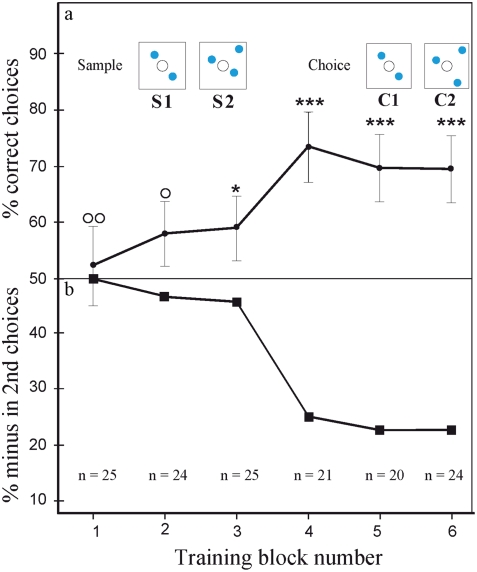
(a) Learning curve for bees trained on a basic delayed match-to-sample (DMTS) task. Each ‘block’ represents two twenty-minute sessions of training (one for each sample S1 and S2). Bees were considered to be trained in this task when their performance reached a stable plateau (approximately 70% correct choices). n denotes number of bees per condition. Error bars show standard error. *** denotes statistically significant difference at p<0.001, * denotes p<0.05. (b) The incorrect second choices of bees in each of the training blocks, following a positive first choice.

Over a period of three days, a group of approximately 20 bees were able to learn the basic DMTS paradigm, where they had to match one of two choice patterns to a previously encountered sample pattern (See the experimental apparatus in Fig. 1). In particular, bees could choose a pattern of two or three blue dots that exactly matched the sample pattern in every way, in order to obtain a sugar reward. The ANOVA tests revealed that the data collected across all blocks and across all bees were homogeneous (p>0.05). The exact p values for each block are summarised in Supporting Table S1. Fig. 2a shows the learning curve of the experimental bees in the six training blocks; performance is seen to plateau at approximately 70% correct choices after the 4^th^ block. Fig. 2b shows the percentage of incorrect second choices following a positive first choice. The percentage of incorrect second choices of the 1^st^ block was 50.0%; this declined to a low 24% by the last block.

### Transfer test on patterns containing dots in randomised orientations

**Figure 3 pone-0004263-g003:**
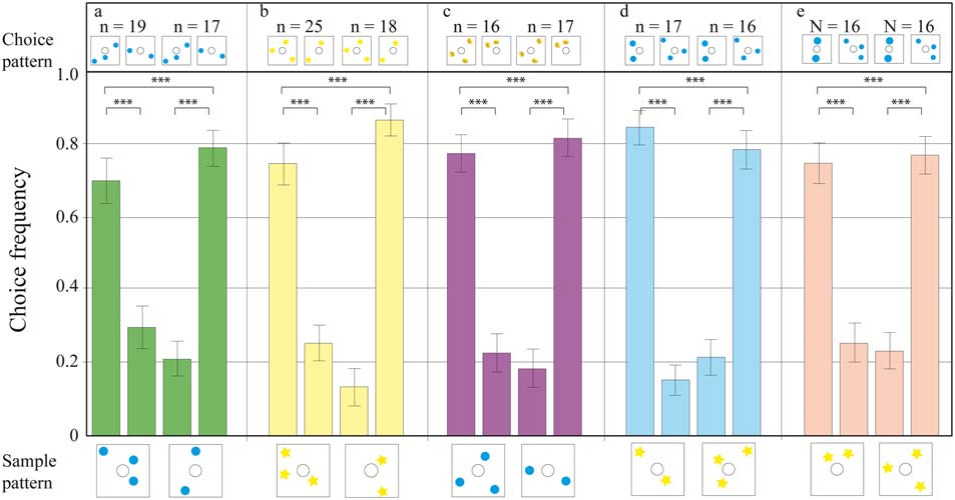
Results of transfer tests with various pattern configurations. The pattern below each pair of bars is the sample and that above each bar is the choice pattern; the y-axis gives the choice frequency. The data represent the pooled first choices (from each foraging trip) of individual bees. (a) The configuration of dots on the sample and choice patterns is randomised. (b) The blue dot patterns in (a) are replaced with yellow stars, to see if bees can transfer their matching ability to different, unknown stimuli. (c) The sample and choice patterns are composed of different elements. (d) The choice patterns are modified so that the total area of the elements is equal. (e) The choice elements are modified so that the total edge length of the elements is equal. n = number of bees per condition. Error bars show standard error. *** denotes statistically significant difference at p<0.001, ** denotes p<0.01, * denotes p<0.05 and ○ denotes p>0.05.

Once the bees' performance in the basic DMTS task had stabilised, they were presented with new patterns, in which the configuration of dots was randomised. There were 19 bees in the 1^st^ half of the transfer test when the sample was three blue dots, and 17 bees in the 2^nd^ half of the transfer test, when the sample was two blue dots (as denoted in Fig. 3a). An individual bee visited the apparatus during a transfer test for four times on average (one visit per configuration). The bees were able to carry out this more difficult task, and attained a score of 70% (significantly different to 50%, p<0.01) for the three-dot-sample, and 79% (significantly different to 50%, p<0.001) for the two-dot-sample. The performance was significantly reversed after the sample pattern was swapped from the two-dot sample to the three-dot-sample (p<0.001, Fig. 3a). The same notations are used in all other figures. This experiment gave the first indication that the trained bees might be using the number of elements in the visual arrays as a cue to perform the matching task.

### Transfer tests with novel stimuli

We then tested whether the same trained bees could transfer the rule “match the number of items” to a totally novel set of stimuli. Once again, when the bees were shown a sample pattern containing two stars, for example, they were able to convincingly match it with a choice pattern also containing two stars (Fig. 3b). This experiment was repeated with further sets of novel stimuli, such as two versus three yellow lemons, with much the same result (see Supporting Figure S1a and Supporting Table S2 for statistical analysis). Then, an additional level of abstraction was introduced, by making the elements of the sample and choice patterns different. Now, the bees encountered a sample pattern of three blue dots, for instance, which they had to match to a choice pattern composed of three yellow lemons, again in random configurations. Here too, the bees performed remarkably well, using the number of items to identify the matching, rewarded pattern (Fig. 3c). Reversing the order of the patterns, i.e. yellow lemons as the sample and blue dots as the choice patterns, did not affect performance (See Supporting Figure S1b).

### Control tests for lower-order cues

To control for the possibility that the bees could be using cues such as the edge lengths or combined areas of the visual items, we presented them with stimuli where these cues, in the sample and choice patterns, had been equalised. The bees were still able to choose the pattern with the right number of dots, even when the areas (Fig. 3d) and edge lengths (Fig. 3e) of the choice patterns were the same.

### Transfer tests with novel numerosities

**Figure 4 pone-0004263-g004:**
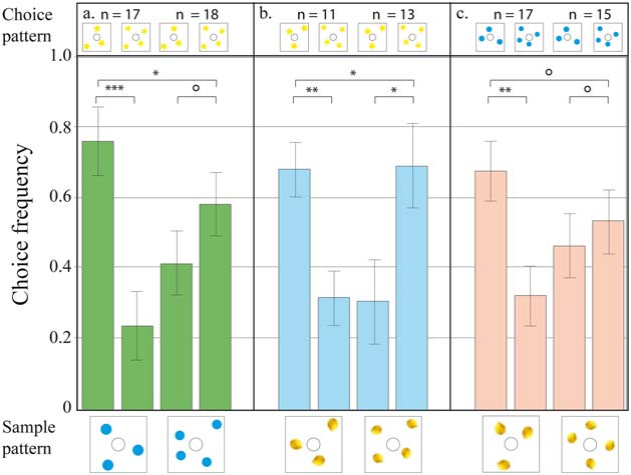
Results of transfer tests to determine the trained bees' ability to discriminate between three and four without any prior training on patterns with four elements. The notations used here are the same as those in Figure 3.

**Figure 5:**
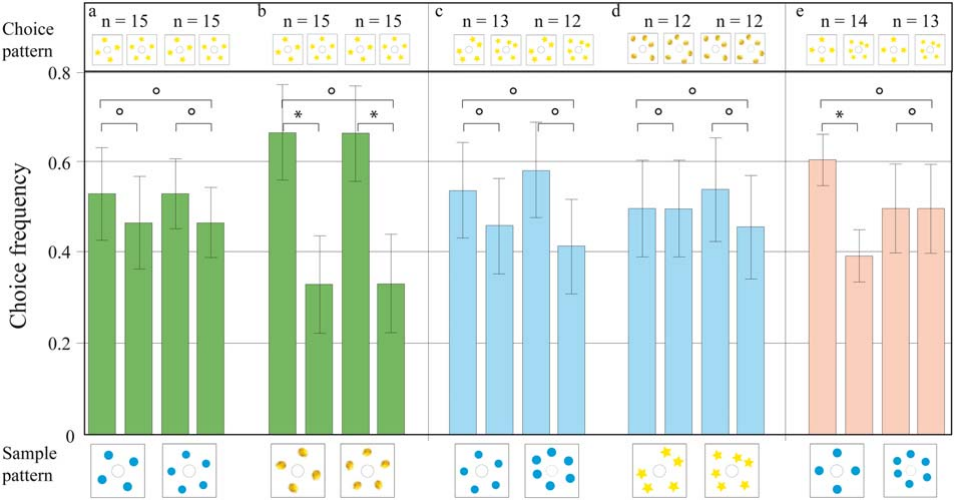
Results of transfer tests to determine our trained bees' ability to discriminate between patterns containing higher (unknown) numbers of elements. (a–b) Bees trained to discriminate between two and three are tested on patterns with four and five elements. (c–d) Discrimination by the same set of bees between five and six. (e) Discrimination by the same set of bees between four and six. The notations used here are the same as those in Figure 3.

Next, we investigated if the bees could transfer their ability to discriminate between two and three, to arrays of three and four items, the latter being a value they had not previously encountered during the experiment. The bees could successfully carry out a three-to-three match, when the competing stimulus contained four elements (Fig. 4a to c). However, they were not able to consistently do a four-to-four match, at a level significantly above chance, when the competing stimulus contained three elements. Thus, there seemed to be a definite limit to their ability to extrapolate to higher numerosities: their performance in discriminating four versus five, five versus six and four versus six was also not above chance in all tests. The bees were not able to decide for a choice pattern according to the numerosity of the sample (Fig. 5a to e).

### Control test for illusory contours

**Figure 6:**
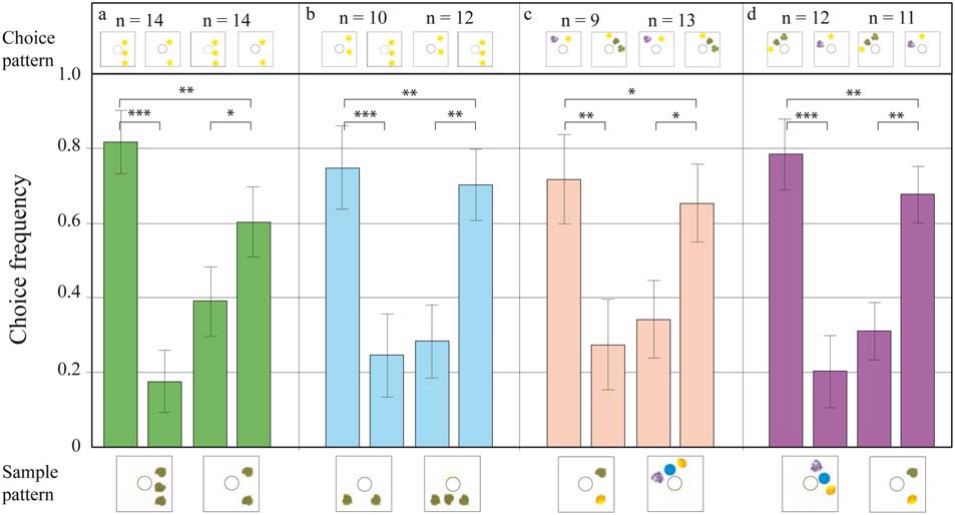
Results of tests to determine our trained bees' ability to discriminate between patterns containing elements arranged in straight lines of equal length. (a) The sample and choice patterns are both oriented vertically. (b) The sample and choice patterns have different orientations. (c) and (d) the sample and choice patterns have different orientations, and are composed of different elements. Note the ‘misdirecting’ cues: the purple flower in (c) and the dark-green leaf in (d). The notations used here are the same as those in Figure 3.

Since bees are able to detect illusory contours [19], we carried out another series of control experiments where the elements in a visual array were always arranged in straight lines of equal length (Fig. 6a to d). This prevented the bees from using the overall shape described by the elements (i.e. a triangle versus a straight line) to carry out the matching task. Once again the bees were able to match the right number of elements, even in mixed arrays (i.e. when the arrays were composed of mixtures of elements, and there were no elements in common between the sample and choice patterns) (Fig. 6c and 6d).

### Control tests for olfactory cues

In order to exclude the possibility that olfactory cues of the feeder were influencing the bees' decisions, we carried out an additional control experiment (See Material and methods). When the three dot sample was presented, the bees preferred the three dot choice pattern (0.78±0.15 of the decisions), at a level significantly different to random choice (n = 9, total 17 visits, p<0.001). When the two dot sample was presented, they significantly preferred the two dot pattern. The choice frequency of 0.74±0.10 for the two dot pattern is also significantly different to random choice (n = 11, total 23 visits, p<0.001). The bees significantly reversed their preference when the sample pattern changed (n = 12, total 40 visits, p<0.001). The use of new maze cylinders and the absence of a feeder behind the correct pattern did not impair the bees' ability to solve the task, showing that olfactory cues do not play a role in the bees' decision making in our experimental paradigm.

## Discussion

Our results clearly demonstrate that honeybees can use the number of elements in a visual pattern, to match a choice stimulus with a sample stimulus in a DMTS paradigm. First, we were able to confirm earlier findings that bees are able to learn the abstract concept of ‘sameness’ [9]. Using this as a starting point, we then tested bees on progressively more challenging sets of stimuli, where only the number of elements in each stimulus was kept constant. While the first training experiment only required the bees to match patterns that were identical in every respect, we subsequently showed that bees could transfer the matching rule even to stimuli where the elements (the blue dots) were in different, random orientations. They were able to match stimuli which contained novel elements, also in random orientations, and to match sample and choice stimuli that contained different elements. Our control experiment for illusory contours confirms that the bees were not using the overall shape described by the elements as a cue.

Given any one set of sample and choice patterns from our experiment, it would be quite reasonable to suggest alternative hypotheses for the bees' performance: bees could indeed be using lower-order visual cues, or relying on accidental features shared by the sample and rewarded choice pattern. After all, ants can use ambient light levels within a nest cavity to estimate the number of nest entrances, while evaluating a potential residence [20]. However, our protocol involved training a single group of approximately 20 bees on a standard DMTS task, and later testing them sequentially with the entire set of novel patterns, where only the number of elements was kept constant (See Supporting Figure S2 for a list of all the patterns used). The entire experiment was repeated twice with different sets of bees. Over the course of the testing procedure, a bee that had successfully matched three blue dots to three yellow lemons (arranged in random configurations) might, 40 minutes later, be required to match two green leaves to two yellow stars (arranged in straight lines of equal length). If the bees were employing lower-order or accidental features, it would have had to re-teach them for each new set of patterns, which would have taken a few trips to the apparatus in each condition. Instead, our bees were mostly able to solve such tasks immediately, as evidenced by the first-choice data of individual bees presented in Supporting Tables S3 a & b and S4.

We also tried to make the sample and rewarded choice patterns as dissimilar as possible in terms of element configuration, and also deliberately tried to induce the bees to choose the wrong pattern in some experiments. Thus, in Fig. 6c, the three-element sample pattern and the incorrect two-element choice pattern both contain a purple flower, while the dark-green leaf in the two-element sample pattern (Fig. 6d) serves the same purpose. Still, the majority of bees ignored such obvious (misdirecting) cues, and chose the pattern with the right number of elements. Finally, our observation that bees can adapt well to novel visual stimuli (in terms of element type and orientation) containing the same number of elements, but not to those containing a novel number of elements, indicates that element number was a salient cue.

**Figure 7:**
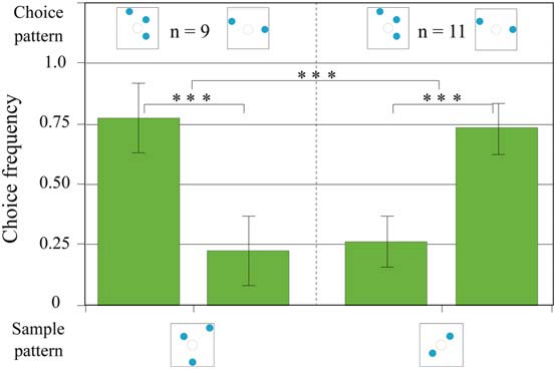
Results of the control tests for olfactory cues. The notations used here are the same as those in Figure 3.

The presence of a feeder during all stages of testing could be considered a departure from a standard memory testing protocol. The advantages of unrewarded testing include the certain exclusion of olfactory cues from the feeder, and the prevention of learning during the tests. Such testing conditions are essential only when bees are trained to a simple task, where individual bees have to go through the transfer test only once. However, as mentioned above, we wanted to ensure that the same group of trained bees kept visiting the maze throughout the duration of the experiment, *i.e.* over the complete series of transfer tests. Had we put them through unrewarded tests, many of the trained bees would have lost their motivation after a few attempts, and stopped visiting the apparatus. After all, in our experimental paradigm, the experience of an unrewarded test, where the bee makes a correct decision but doesn't find a feeder behind the correct choice pattern, is similar to the punishment for making a wrong decision, and thus equivalent to negative training. One could argue that bees might not be able to solve the task without the help of olfactory cues, although these non-visual stimuli alone are not sufficient to support correct choices. However, as mentioned later in this section (‘The absence of olfactory cues’), it has been conclusively shown that the presence of a feeder during a test does not lead to false positives in the bees' choice data. If olfactory cues did exist, the bees should have found the feeder in the case of our four vs five or four vs six dot experiments as well. In the control experiment for olfactory cues, a new set of bees was trained to the basic DMTS task, and then tested in fresh maze chambers without a feeder. The data show that the bees are able to choose the correct number of elements according to the sample pattern without the presence of a feeder in the final chamber (see Methods section and Fig. 7 for details). In Fig. 2, we show that the percentage of incorrect second choices following a positive first choice in the 1^st^ block was 50.0%, which declined to a low 24% by the last block. This unequivocally supports the absence of olfactory cues at the feeder. In addition, we made the following observation at the end of the complete series of transfer tests, in which i) the two choice patterns and the sample pattern were identical; ii) there was a feeder with sugar water behind one choice pattern and a feeder with only water behind the other choice pattern; and iii) the positions of the two feeders were swapped after 5 min., which is half the normal testing period. The visiting frequency at the two feeders during the 10 min. observation period was 20: 17 (the feeder with sugar water to the feeder with water). There is no significant difference from random choice level (Chi = 0.003, P>0.90).

Moreover, it is highly unlikely that the bees' performance is somehow predicated on the additional training they might receive by the attainment of a reward during the testing procedure. The reliable learning of a pattern takes about 15–20 rewarded exposures (*e.g.* [21]), but in our experiment, the average bee would only be able to visit the feeder a maximum of four times during a test. In any case, any learning that did take place during our tests would only be reinforcing the basic DMTS rule of ‘match the right choice pattern to the sample’; this is in no way contrary to the aims of our study. Even if the bees learned the details of a particular pattern, that would also not invalidate our data, as 1) only the first choice of a bee per test condition was analysed, and 2) a radically different novel test pattern would be presented to the bees in the following test condition (see Methods and above). The analysis of incorrect second choices (Fig. 2b) showed that in the early stages of training, up to 50% of bees were choosing the negative pattern on their second visit, even though their first choice was correct. This is more evidence that the bees did not use scent as a cue. The frequency of this type of error declined with training.

As mentioned in the Introduction, an estimation of relative numerical quantity could be extremely useful to foraging honeybees: combining information on the degree of stomach distension along with the number of flowers visited on a foraging trip could provide bees with an index of the profitability of a food source. Honeybees can recognise images of complex natural scenes [22], and may be able to use them as potential landmarks. The number of landmarks encountered on a foraging trip, or found near the hive, could be useful in navigation [15], [16]. Number generalisation could also help in estimations of the number of blossoms on a branch and/or, the number of bees on a blossom, thereby allowing a new forager to decide whether to forage at that location, or look for a new one. It has been suggested that both duration and numerosity may be represented mentally in animals through the same mental magnitudes, i.e. through real numbers [23]. As the ability to measure time intervals was recently demonstrated in the bumblebee [24], there is a pleasing sense of symmetry in our demonstration of the honeybee's ability to distinguish between visual arrays of two and three elements, using only element number as a cue, and to even transfer this ability to discriminate between completely novel stimuli containing three and four elements.

The only unifying feature of all the patterns used in our tests was that they contained the same number of elements that the bees had initially been trained on. We are not proposing here that the bees were ‘counting’, *sensu stricto* [25], that they possess mathematical competence [26], or that they were able to order the abstract concepts of ‘two’ and ‘three’ on a scale of magnitude [27]. We conclude that the bees are able to make generalisations about patterns based on the number of elements, and transfer this ability to discriminate between two and three to new situations. Our most intriguing result was their ability to match three-element stimuli, when the competing stimulus was a four-element pattern. However, their performance in the four-five, five-six and four-six comparisons was not above chance. This last result further supports our conclusion that the bees were indeed using element number to decide which chamber of the y-maze to enter: if the bees had been using lower-order cues such as edge length, dot density, or the area of the dots or background (or even odour cues), one would have expected them to perform just as well in this condition, as they had in previous conditions. Instead, the bees performed well only when at least one of the patterns in the decision chamber contained a number on which they had been previously trained (i.e., two or three). As early as 1871, Jevons proposed that the maximum number of items that a human could accurately estimate with just a moment's exposure lay ‘half-way between’ four and five [28]. More recently, Cowan (2000) presented an impressive amount of evidence to support his claim that, due to attentional limitations, the number of items that humans can hold in their short-term memory and subsequently recall is four, or very close to it [29]. Since the DMTS paradigm is partly a test of the honeybees' short-term memory (which displays temporal decay [17]), it is possible that the mechanisms elucidated by Cowan have a bearing on our results. There is, in addition, evidence that human infants rely on mechanisms of object-based attention and short-term memory to represent small numbers of objects: they can discriminate arrays containing 1, 2, or 3 objects, but fail with arrays greater than 3 [30]. This upper limit also seems to apply to rhesus monkeys [31]. However, these last results are not directly comparable to ours, as the former represent the spontaneous choices of experimental subjects, whereas the latter are the consequence of extensive training. Moreover, the observation that our bees could distinguish between two and three, but not four and six, indicates that performance was not dictated by the ratio difference in set sizes, which, in contrast, seems to be the case in human infants, at least for large numerosities [32].

Another intriguing finding from our study is the improved performance of our trained bees in the transfer tests, in comparison to the last sessions of training on the basic DMTS task. We hypothesize that the reason for this effect is the novelty of the test patterns – after three days of training on the same set of visual patterns, the bees were presented with patterns of increasing novelty in the transfer tests. We noticed that by the last stages of training, bees would often proceed past the sample and choice patterns, and into the (correct or incorrect) reward chamber with only a cursory scan of the patterns. When presented with novel test patterns, however, bees would regularly spend more time scanning them, and were frequently seen to approach each element in a pattern, before passing into the next chamber. Chittka et al. (2003) have shown that the more time an individual bumblebee invests in making a decision, the more accurate are its responses [33]. In addition, Heisenberg et al. (2001) have reported the phenomenon of ‘selective attention’ in flies, with tethered *Drosophila* able to preferentially attend to one of two competing stimuli [34]. Van Swinderen (2007) showed that *Drosophila* reacted to novel visual patterns, and that mutants deficient in genes implicated in short-term memory also suffered from attention deficits [35]. It is possible that our bees, too, not only attend to novel stimuli for longer, but also achieve improved memory scores as a result.

Recent research has revealed that perceived numerosity is susceptible to adaptation, in the same way as the primary visual properties of a scene, such as colour, contrast, size, and speed [36]. Numerosity can therefore be considered an independent primary visual property which, as our results demonstrate, can also be apprehended by honeybees. Our study therefore suggests a fruitful line of investigation for the future, as the limits of this and other invertebrates' cognitive abilities remain to be determined.

### The absence of olfactory cues

A possible role of olfactory cues as a confounding factor in experiments such as ours has been excluded here as in former experiments.Van Hateren et al. (1990) [37] and Zhang et al. (1996, 1999, 2004 and 2005) [8], [10], [17], [37], [38] have carried out tests to address this very question, and found that the presence of a hidden feeder behind one of a set of identical choice patterns (in a similar, but more elaborate y-maze setup in the 1996 study, and in a maze much like the present one in the 2005 study) does not in the least influence the probability of a bee choosing that pattern. To address this question, we have conducted an additional experiment to control for olfactory cues. A group of bees was trained to the basic DMTS task with visual patterns containing two and three blue dots, and then tested on patterns with randomised dot orientations in fresh maze chambers and without a feeder behind the correct pattern. The bees were still able to solve the task (see Results section and Fig. 7 for details). These data also show that the presence of a hidden feeder does not influence the bees' choice of a particular pattern in our experimental setup.

The feeder in our experiments was found and visited by bees, which presumably would have left scent marks in that choice chamber. However, this did not make that chamber any more attractive to subsequent bees.

When honeybees opened their Nasonov gland in our experiments, this was clearly visible to the experimenters. This rarely happened at all, and if it did, we removed the bees from the maze. If the feeder had carried any scent, from the Nasonov pheromone or otherwise, the bees would have been able to solve any task we presented to them, no matter what item numbers were visible on the visual patterns. However, they were not able to do a 4 to 4, 5 to 5 or 6 to 6 match, thus demonstrating the complete absence of olfactory cues.

## Materials and Methods

### Basic training

A group of approximately 20 bees was trained in a modified y-maze apparatus to perform a basic DMTS task [9] with identical patterns of two versus three blue dots. Briefly, when a bee entered the apparatus, it encountered a sample pattern, say two blue dots, which it had to retain in its working memory. The bee had to then fly through a 1-meter long tunnel and then into a decision chamber, where it was presented with two choice patterns, only one of which was identical to the sample. The other pattern was composed of three blue dots. The bee had to choose the matching pattern (two dots), to obtain a reward of sugar solution from a hidden artificial feeder. A bee making an incorrect decision was released from the maze and allowed to try again. However, only the first-trial data for each bee were used. The positions of the choice patterns were swapped every ten minutes, to prevent the bees from developing a side preference. Thus each sample pattern was presented for twenty minutes during training. Every time the positions of the choice patterns were exchanged, they were also rotated by 180°, as was the sample pattern. Once training was completed on a particular sample (say, two dots), it was replaced with the competing sample (three dots); this sample was also presented for two ten-minute sessions. In all, each pair of competing patterns was presented to the bees in four orientations: two dots (upright), two dots (rotated), three dots (upright), and three dots (rotated). Training, including pre-training and training proper went on for a total of three days, by which time the bees were able to consistently solve the DMTS task. During the training proper, baffles behind the entrances of the two choice chambers (See Fig. 1) completely prevented the bees from viewing the feeder in the reward chamber from the decision chamber. The learning curve was acquired during this period.

### Testing and data collection

A rewarded feeder was present in the ‘correct’ chamber at all times during testing. Testing was carried out in shorter, 5-minute blocks, as only the first choices of the bees (per test condition) were of interest to us. This procedure also had the effect of minimizing any additional learning that might have taken place during the test sessions. Every time the positions of the choice patterns were swapped, they were also rotated by 180° to ensure that the bees were not learning a particular configuration of elements (See [17] for further details). Non-choices, where a bee enters the choice chamber, but is unable to decide on a pattern for an extended period of time, occurred frequently in the early stages of training, but had ceased to be a problem by the time testing commenced. The experiment was halted for 30–40 minutes between each transfer test. During these breaks, another feeder, with a dilute sugar solution, was provided at the entrance of the tunnel (which was otherwise blocked). This procedure, combined with frequent transfers to novel sets of stimuli, improved the bees' performance, compared to a regime of prolonged, uninterrupted training on a single set of stimuli. Note, for example, that the bees frequently performed better in the transfer tests with novel stimuli than in the last two sessions of the learning curve.

As the y-maze had only two reward chambers, we took pains to randomize the position of the starting chamber in each transfer test. This prevented the bees from learning a rule like ‘go to the left chamber at the start of each test’.

### Controlling the number of bees within the apparatus

We were careful, during the transfer tests, to limit the number of bees in the choice chamber to one at a time. This is important, because bees readily follow each other within the confined space of the apparatus, and can also distract one another during the decision-making process. To a large extent our experimental design ensured that the y-maze was never visited by a surplus of bees during any given transfer test. The extended breaks between each transfer test, combined with the weaker sugar solution offered at the maze entrance during these breaks, had the effect of temporarily reducing the traffic of our 20 trained bees between the maze and the hive. Moreover, during the occasions when more than one bee did enter the decision chamber, the experimenter would open the lid of the chamber, and let the excess bees out of the maze. These bees would then have to return to the maze entrance to try again. Thus we ensured that bees were making independent decisions.

### Control tests for olfactory cues

In order to exclude the possibility that olfactory cues were influencing the bees' decisions, we carried out an additional control experiment. A group of bees was trained specially for this purpose. The bees were trained to solve a basic DMTS task with a set of patterns containing two and three blue dots that were the same as used in the previous training (Fig. 2a), and were then, after they had reached a high plateau, tested on new patterns with randomised two and three dot configurations. During the transfer tests, the three cylinders of the maze apparatus (Fig. 1) were replaced with fresh ones, and no reward was present in the end cylinders. The testing period was kept short (2–3 min), to make sure that each bee would encounter the unrewarded transfer test situation only once, since it is similar to the punishment for making a wrong choice for the bee, and thus leads to negative learning. Each of those tests was followed by a long training period to keep the bees motivated to visit the maze.

### Statistical analysis

We performed ANOVA using the statistical software SYSTAT for checking the homogeneity of the data (Systat Software, Richmond, CA). Next, the performance of each bee was evaluated separately, by pooling its first choices. The mean choice frequency was calculated as follows: the first choice of each bee in a given test condition, if correct, was scored 1, and if wrong, was scored 0. Each bee provided one data point in the first test configuration (with the patterns upright), and then another in the second test configuration, when the feeder was moved into the other chamber, and the patterns were rotated by 180°. A bee could therefore achieve an average score of 0%, 50% or 100% in a transfer test. The average scores of all bees involved in the test were averaged for an overall indication of performance. The Student t-test was used to determine whether performance was significantly better than random choice. Two types of Student t-tests were performed: the first type of test was to check whether the bees made decisions according to the sample pattern, namely whether their performance was significantly different from random choice; the second type of test was to check whether the bees reversed their preference after the sample was changed.

## Supporting Information

Figure S1 Results of transfer tests with further sets of novel stimuli. (a) The yellow stars in Fig. 3b are replaced with yellow lemons; (b) Reversing the order of the patterns in Fig. 3c, i.e. yellow lemons as the sample and blue dots as the choice patterns. The notations used here are the same as those in Figure 3.
(1.52 MB TIF)

Figure S2 All sample and choice patterns used in the learning tests and various transfer tests. Each group of bees was tested on a large number of patterns, both in the orientation shown above, as well as rotated 180.
(2.35 MB TIF)

Table S1 In each block Bees denotes the variance inherent in the performance score plus variance attributed to an individual bee's variation; Error denotes only the variance inherent in the performance score; d.f. lists degrees of freedom for the specified conditions; F-ratio is the Mean-Square for Bees divided by the Mean-Square for Error. The P value is probability of exceeding the F-ratio when the group means are equal.
(0.04 MB DOC)

Table S2 summarises the details of the student t tests for Figures 2–5 and Figure S1. Test Type 1 was to check whether the bees made the decisions according to the sample pattern, namely whether their performance was significantly different from random choice; Test Type 2 checked whether the bees reversed their preference after the sample was changed listed under Reversing preference tests. For each test, student t, df (degree of freedom) and p values are given in the table.
(0.05 MB DOC)

Table S3 A & B. Individual performance records of three bees (from a group of about twenty) trained in an experiment performed in December 2006. The first choice of each bee within a ten-minute testing block is shown (+ correct choice, − incorrect choice). A blank cell indicates that the bee did not visit the apparatus during that block. These bees were chosen as they were involved in all steps of the transfer tests. 3+ and 2+ indicate the number values of the rewarded patterns, while 0 and 180 indicate pattern orientation within a ten-minute block (as each set of sample and choice patterns was tested in two orientations). R and L mean that the reward was in the right or left arm of the y-maze. Each pair of 3+ and 2+ columns represents a different set of novel test stimuli. The choice patterns and the sample pattern for each set of the tests are shown respectively above and below the choice performance.
(0.55 MB PDF)

Table S4 Individual performance records of five bees (from a group of twenty) trained in an experiment performed in April 2007. The notations used here are same as in Table S1.
(0.35 MB PDF)
