# Supplementary figures and images for: Number-Based Visual Generalisation in the Honeybee
Source: PLoS One. 2009 Jan 28;4(1):e4263. doi: 10.1371/journal.pone.0004263 (PMC2629729; doi:10.1371/journal.pone.0004263)

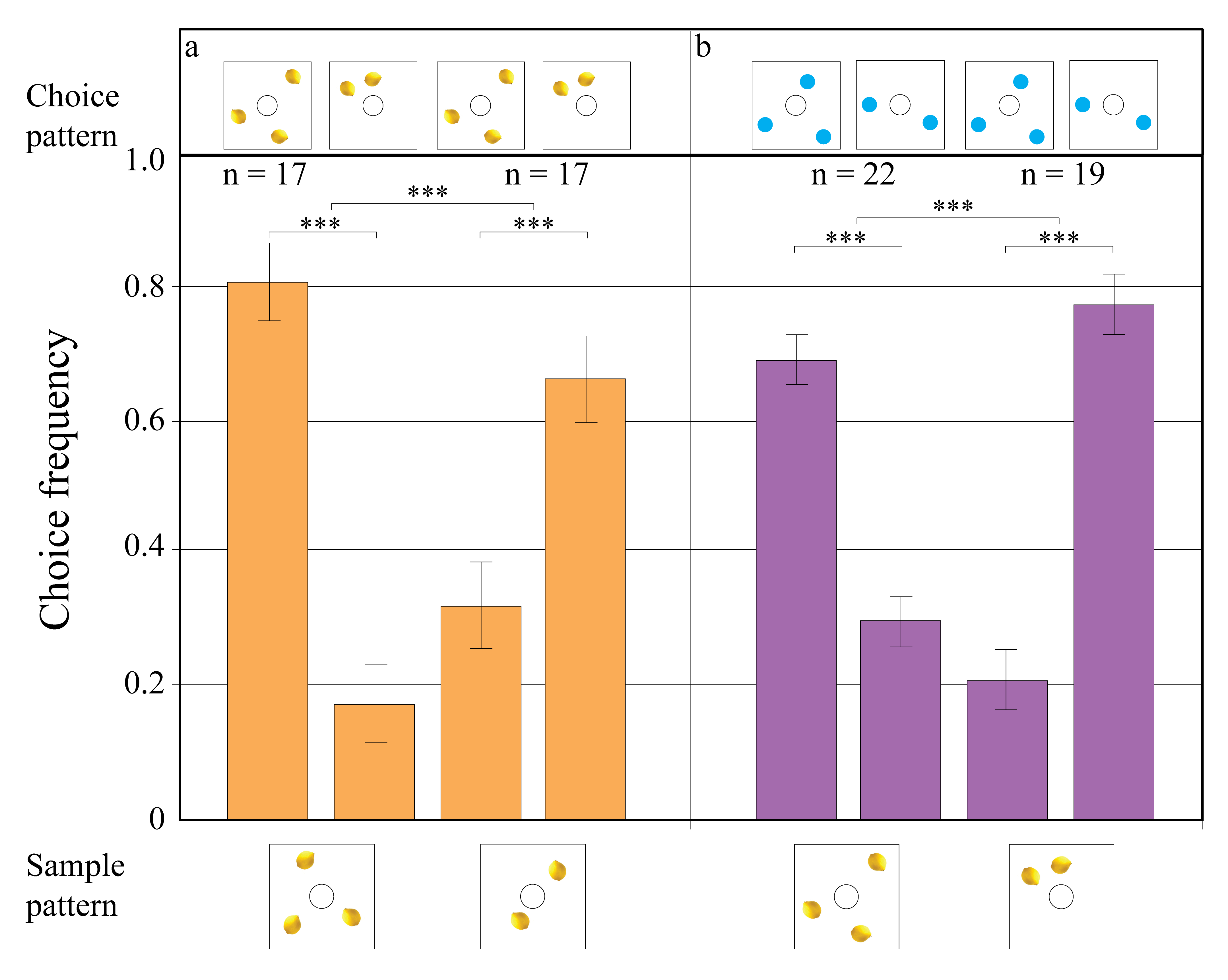

Supplement: Figure S1 — Results of transfer tests with further sets of novel stimuli. (a) The yellow stars in Fig. 3b are replaced with yellow lemons; (b) Reversing the order of the patterns in Fig. 3c, i.e. yellow lemons as the sample and blue dots as the choice patterns. The notations used here are the same as those in Figure 3. (1.52 MB TIF) [file pone.0004263.s001.tif]

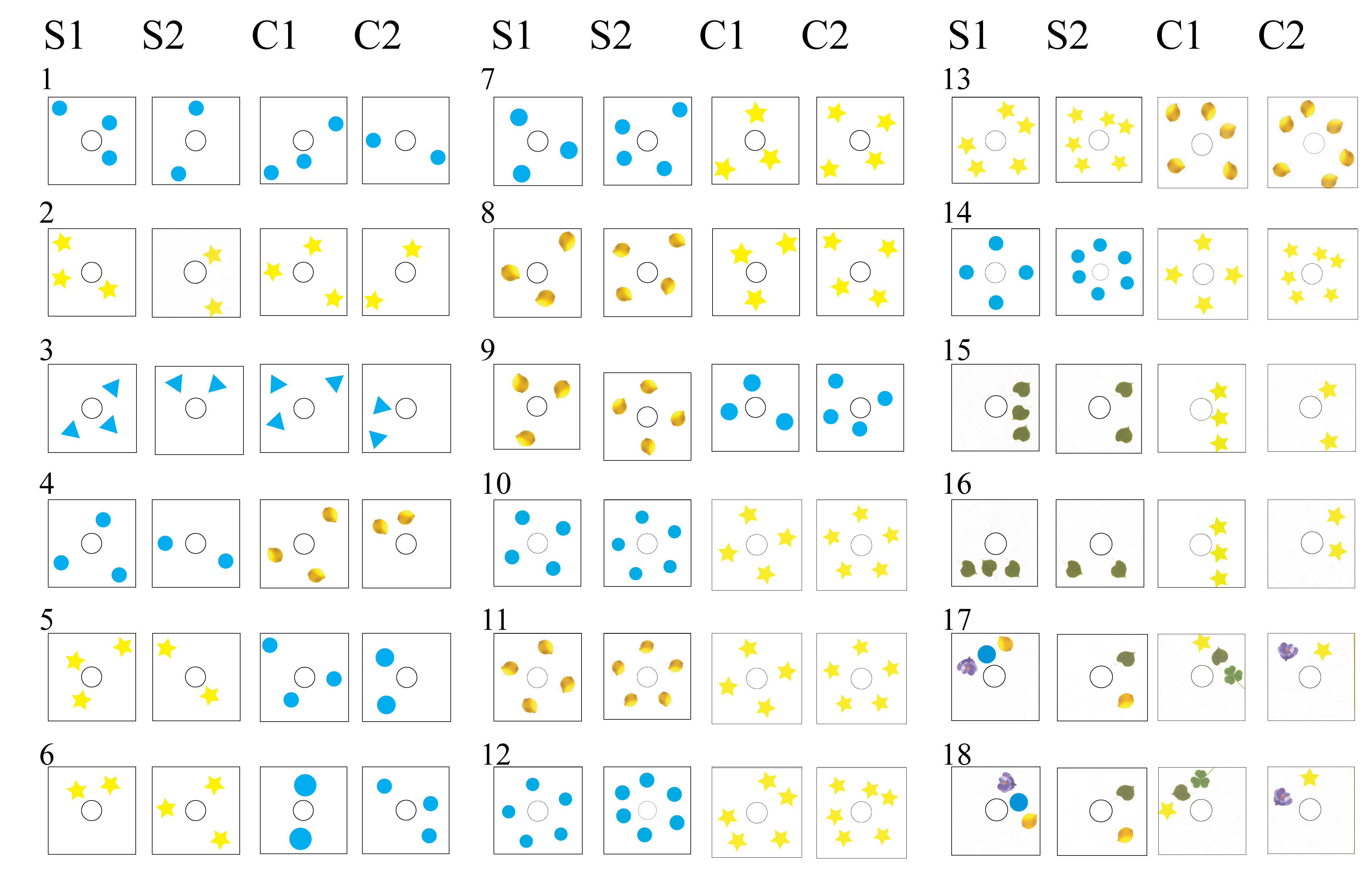

Supplement: Figure S2 — All sample and choice patterns used in the learning tests and various transfer tests. Each group of bees was tested on a large number of patterns, both in the orientation shown above, as well as rotated 180. (2.35 MB TIF) [file pone.0004263.s002.tif]
