# Supplementary material for: Number-Based Visual Generalisation in the Honeybee
Source: PLoS One. 2009 Jan 28;4(1):e4263. doi: 10.1371/journal.pone.0004263 (PMC2629729; doi:10.1371/journal.pone.0004263)
Supplement: Table S1 — In each block Bees denotes the variance inherent in the performance score plus variance attributed to an individual bee's variation; Error denotes only the variance inherent in the performance score; d.f. lists degrees of freedom for the specified conditions; F-ratio is the Mean-Square for Bees divided by the Mean-Square for Error. The P value is probability of exceeding the F-ratio when the group means are equal. (0.04 MB DOC) [file pone.0004263.s003.doc]

Analysis of Variance for blocks during learning

| Block No. | Source | Sum-of squares | d.f. | Mean-square | F ratio | *p* |
| --- | --- | --- | --- | --- | --- | --- |
| Block 1 | Bees | 5.358 | 22 | 0.244 | 0.965 | 0.518 |
|  | Error | 15.148 | 60 | 0.252 |  |  |
| Block 2 | Bees | 5.699 | 20 | 0.285 | 1.226 | 0.271 |
|  | Error | 12.315 | 53 | 0.232 |  |  |
| Block 3 | Bees | 5.365 | 21 | 0.255 | 1.023 | 0.452 |
|  | Error | 14.733 | 59 | 0.250 |  |  |
| Block 4 | Bees | 2.442 | 15 | 0.163 | 0.674 | 0.795 |
|  | Error | 11.106 | 46 | 0.241 |  |  |
| Block 5 | Bees | 2.289 | 15 | 0.153 | 0.651 | 0.816 |
|  | Error | 10.311 | 44 | 0.234 |  |  |
| Block 6 | Bees | 4.599 | 17 | 0.271 | 1.257 | 0.255 |
|  | Error | 11.839 | 55 | 0.215 |  |  |
