# Supplementary material for: Number-Based Visual Generalisation in the Honeybee
Source: PLoS One. 2009 Jan 28;4(1):e4263. doi: 10.1371/journal.pone.0004263 (PMC2629729; doi:10.1371/journal.pone.0004263)
Supplement: Table S2 — summarises the details of the student t tests for Figures 2– 5 and Figure S1. Test Type 1 was to check whether the bees made the decisions according to the sample pattern, namely whether their performance was significantly different from random choice; Test Type 2 checked whether the bees reversed their preference after the sample was changed listed under Reversing preference tests. For each test, student t, df (degree of freedom) and p values are given in the table. (0.05 MB DOC) [file pone.0004263.s004.doc]

**Table S2**

|  | **Test Type 1** | | **Test Type 2** |
| --- | --- | --- | --- |
| **Figure 2** | **Sample 3** | **Sample 2** | **Reversing preference tests** |
| Figure 2a | t=3.4;df=18, p=0.003 | t=5.7,df=16,p=0.0000 | t=6.6, df=34, p=0.0000 |
| Figure 2b | t=4.6,df=24,p=0.0002 | t=7.29,df=17,p=0.0000 | t=8.1, df=41, p=0.0000 |
| Figure 2c | t=5.5,df=15,p=0.0000 | t=6.3,df=16,p=0.0000 | t=7.9,df= 31, p= 0.0000 |
| Figure 2d | t=8.01,df=16,p=0.0000 | t=5.45,df=15,p=0.0000 | t=9.3, df=31, p= 0.0000 |
| Figure 2e | t=4.3, df=15, p=0.0006 | t=4.9, df=15, p=0.0002 | t=6.55,df=30, p=0.0000 |
|  |  |  |  |
| **Figure 3** | **Sample 3** | **Sample 4** | **Reversing preference tests** |
| Figure 3a | t=3.86,df=32,p=0.0005 | t=1.26, df=34, p=0.21 | t=2.60,df =33, p= 0.014 |
| Figure 3b | t=3.39,df=20,p=0.0029 | t=2.25, df=24, p=0.034 | t=2.51, df=22, p=0.020 |
| Figure 3c | t=2.93,df=32,p=0.0062 | t=0.51, df=28, p=0.615 | t=1.68,df=30, p=0.1027 |
|  |  |  |  |
| **Figure 4** | **Sample 4** | **Sample 5** | **Reversing preference tests** |
| Figure 4a | t=0.29, df=14, p=0.78 | t=0.38, df=14, p=0.72 | t=0.00023,df=28,p=0.999 |
| Figure 4b | t=1.6, df=14, p=0.06 | t=1.61,df=14, p=0.06 | t=0.00, df=28, p=1.0000 |
|  | **Sample 5** | **Sample 6** | **Reversing preference tests** |
| Figure 4c | t=0.37,df=12, p=0.78 | t=0.76, df=11, p=0.46 | t=0.81, df=23, p=0.43 |
| Figure 4d | t=0.00, df=11, p=1.0 | t=0.35, df=11, p=0.73 | t=0.25, df=22, p=0.80 |
|  | **Sample 4** | **Sample 6** | **Reversing preference tests** |
| Figure 4e | t=1.96,df=13, p=0.07 | t=0.00, df=12, p=1.00 | t=0.97, df=25, p=0.34 |
|  |  |  |  |
| **Figure 5** | **Sample 3** | **Sample 2** | **Reversing preference tests** |
| Figure 5a | t=3.78, df=13, p=0.002 | t=1.2, df=13, p=0.25 | t=3.4, df=26, p=0.005 |
| Figure 5b | t=2.3, df=9, p=0.004 | t=2.2, df=11, p=0.05 | t=3.2, df=20, p=0.004 |
| Figure 5c | t=1.8, df=8, p=0.10 | t=1.54,df=12, p=0.149 | t=2.3, df=20, p=0.003 |
| Figure 5d | t=3.1, df=11, p=0.01 | t=2.6, df=10, p=0,026 | t=3.8, df=21, p=0.001 |
|  |  |  |  |
| **Figure S1** | **Sample 3** | **Sample 2** | **Reversing preference tests** |
| Figure S1a | t=5.49,df=16, p=0.000 | t=2.6,df=16, p=0.001 | t=5.4,df=32, p=0.000 |
| Figure S1b | t=5.21,df=21, p=0.000 | t=6.3,df=18, p=0.000 | t=8.3,df=39, p=0.000 |
