# Supplementary material for: Number-Based Visual Generalisation in the Honeybee
Source: PLoS One. 2009 Jan 28;4(1):e4263. doi: 10.1371/journal.pone.0004263 (PMC2629729; doi:10.1371/journal.pone.0004263)
Supplement: Table S3 — A & B. Individual performance records of three bees (from a group of about twenty) trained in an experiment performed in December 2006. The first choice of each bee within a ten-minute testing block is shown (+ correct choice, − incorrect choice). A blank cell indicates that the bee did not visit the apparatus during that block. These bees were chosen as they were involved in all steps of the transfer tests. 3+ and 2+ indicate the number values of the rewarded patterns, while 0 and 180 indicate pattern orientation within a ten-minute block (as each set of sample and choice patterns was tested in two orientations). R and L mean that the reward was in the right or left arm of the y-maze. Each pair of 3+ and 2+ columns represents a different set of novel test stimuli. The choice patterns and the sample pattern for each set of the tests are shown respectively above and below the choice performance. (0.55 MB PDF) [file pone.0004263.s005.pdf]

Table S3a

	Transfer 2v3-1				Transfer 2v3-2				Transfer 2v3-3				Transfer 2v3-4				Transfer 2v3-5				Summary		
Choice patterns	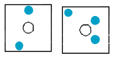		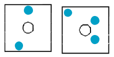		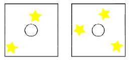		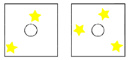		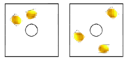		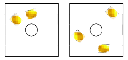		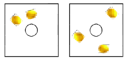		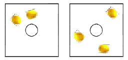		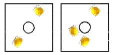		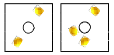				
Rewarded pattern	2+ 0° (R)	2+ 180° (L)	3+ 0° (R)	3+ 180° (L)	2+ 0° (R)	2+ 180° (L)	3+ 0° (R)	3+ 180° (L)	2+ 0° (R)	2+ 180° (L)	3+ 0° (R)	3+ 180° (L)	2+ 0° (R)	2+ 180° (L)	3+ 0° (R)	3+ 180° (L)	2+ 0° (R)	2+ 180° (L)	3+ 0° (R)	3+ 180° (L)	2+	3+	%
Bee 02	+	+	+	+	+	+	+	+	+	-	+		-	+	+	-	+	+	+	+	8+,2-	8+,1-	0.84
Bee 12	+	-	+	+		+	+	+	-	+	-	+		+					+	+	4+,2-	7+,1-	0.79
Bee 1 4	+	+	-		+		+	-	+	+	-	+	+	+	+	+	-	+	+	+	8+,1-	6+,4-	0.74
Sample pattern	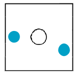		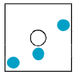		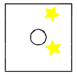		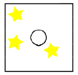		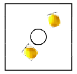		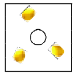		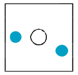		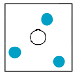		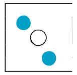		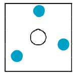				

Table S3b

	Control-same areas					Control-same edges					Transfer 3v4					Transfer 4v5					Control 4v5-1				
Choice Patterns	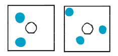		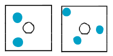			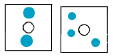		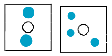			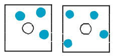		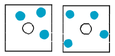			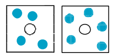		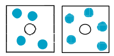			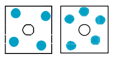		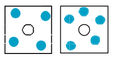		
Reward pattern	2+ 0° (R)	2+ 180° (L)	3+ 0° (R)	3+ 180° (L)	%	2+ 0° (R)	2+ 180° (L)	3+ 0° (R)	3+ 180° (L)	%	3+ 0° (R)	3+ 180° (L)	4+ 0° (R)	4+ 180° (L)	%	4+ 0° (R)	4+ 180° (L)	5+ 0° (R)	5+ 180° (L)	%	4+ 0° (R)	4+ 180° (L)	5+ 0° (R)	5+ 180° (L)	%
Bee 02	+	+	+	+	1.0	+	+	+	+	1.0	+	+	+	+	1.0		-		+	0.5	+	-	+	+	0.75
Bee 12	+	+	-	+	0.75	+	+	+	+	1.0	+	+	-	+	0.75	-		-	+	0.3	-	+	+	+	0.75
Bee 14	-	+	+	+	0.75	+	+	+	-	0.75	+	+	+	-	0.75	-	+	-	+	0.5	+	+	+	-	0.75
Sample pattern	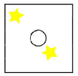		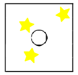			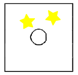		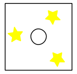			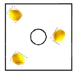		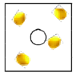			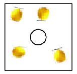		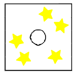			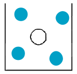		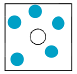		
