# Supplementary material for: Number-Based Visual Generalisation in the Honeybee
Source: PLoS One. 2009 Jan 28;4(1):e4263. doi: 10.1371/journal.pone.0004263 (PMC2629729; doi:10.1371/journal.pone.0004263)
Supplement: Table S4 — Individual performance records of five bees (from a group of twenty) trained in an experiment performed in April 2007. The notations used here are same as in Table S1. (0.35 MB PDF) [file pone.0004263.s006.pdf]

Table S4

	Transfer tests 1				Transfer tests 2				Transfer tests 3				Transfer tests 4				Transfer tests 5				Transfer tests 6			
Choice Patterns	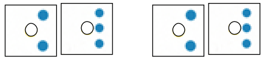				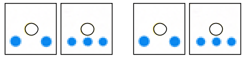				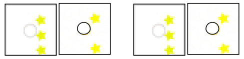				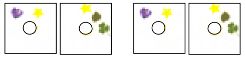				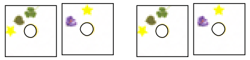				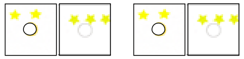			
Rewarded pattern	2+		3+		2+		3+		3+		2+		2+		3		3		2		2+		3+	
	0° (R)	180° (L)	0° (R)	180° (L)	0° (R)	180° (L)	0° (R)	180° (L)	0° (R)	180° (L)	0° (R)	180° (L)	0° (R)	180° (L)	0° (R)	180° (L)	0° (R)	180° (L)	0° (R)	180° (L)	0° (R)	180° (L)	0° (R)	180° (L)
Bee 10	+	+	-	+	+	-	+	+	+	+	+	-			+	+	+	+	+					
Bee 16		+	+	+	+	+	-	+	-	+	-	+				+	+	+					+	+
Bee 21	+	-	+	-	+	+	-	+	+	-	+	+	+	+	+	-	+	+	+	-	+	-	+	+
Bee 31	+	-	+	+	+	+	-	+	+	+	+	+	+	+	-	+	+	+	+	+	+	-	+	+
Bee 60	-	+	+	+	+	+	+	-	+	+	+	-			+		+	-	+	-	+	+	-	+
Sample pattern	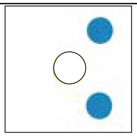		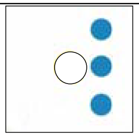		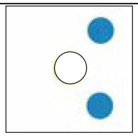		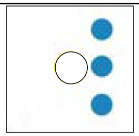		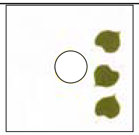		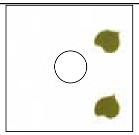		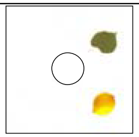		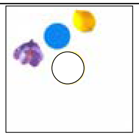		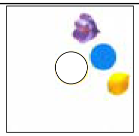		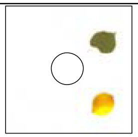		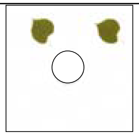		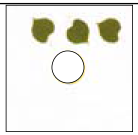	
